# Factors Affecting Quality and Health Promoting Compounds during Growth and Postharvest Life of Sweet Cherry (*Prunus avium* L.)

**DOI:** 10.3389/fpls.2017.02166

**Published:** 2017-12-19

**Authors:** Sofia Correia, Rob Schouten, Ana P. Silva, Berta Gonçalves

**Affiliations:** ^1^Centre for the Research and Technology of Agro-Environmental and Biological Sciences, University of Trás-os-Montes e Alto Douro, Vila Real, Portugal; ^2^Horticulture and Product Physiology, Wageningen University, Wageningen, Netherlands

**Keywords:** growth conditions, quality indicators, phenolic compounds, new preservation technologies, breeding for quality

## Abstract

Sweet cherries are attractive fruits due to their taste, color, nutritional value, and beneficial health effects. Sweet cherry is a highly perishable fruit and all quality attributes and the level of health promoting compounds are affected by growth conditions, picking, packing, transport, and storage. During production, the correct combination of scion × rootstock will produce fruits with higher firmness, weight, sugars, vitamins, and phenolic compounds that boost the fruit antioxidant activity. Orchard management, such as applying drip irrigation and summer pruning, will increase fruit sugar levels and total phenolic content, while application of growth regulators can result in improved storability, increased red coloring, increased fruit size, and reduced cracking. Salicylic acid, oxalic acid, acetylsalicylic acid, and methyl salicylate are promising growth regulators as they also increase total phenolics, anthocyanins, and induce higher activity of antioxidant enzymes. These growth regulators are now also applied as fruit coatings that improve shelf-life with higher antioxidant enzyme activities and total phenolics. Optimizing storage and transport conditions, such as hydro cooling with added CaCl_2_, chain temperature and relative humidity control, are crucial for slowing down decay of quality attributes and increasing the antioxidant capacity. Application of controlled atmosphere during storage is successful in delaying quality attributes, but lowers ascorbic acid levels. The combination of low temperature storage in combination with modified atmosphere packaging (MAP) is successful in reducing the incidence of fruit decay, while preserving taste attributes and stem color with a higher antioxidant capacity. A new trend in MAP is the use of biodegradable films such as micro-perforated polylactic acid film that combine significant retention of quality attributes, high consumer acceptability, and a reduced environmental footprint. Another trend is to replace MAP with fruit edible coatings. Edible coatings, such as various lipid composite coatings, have advantages in retaining quality attributes and increasing the antioxidant activity (chitosan) and are regarded as approved food additives, although studies regarding consumer acceptance are needed. The recent publication of the sweet cherry genome will likely increase the identification of more candidate genes involved in growing and maintaining health related compounds and quality attributes.

## Introduction

Sweet cherry (*Prunus avium* L.) is one of the most popular table fruits. In 2014, the estimation of the world total cherry production was 2.294 thousand metric tons, with the top three producing countries (Turkey, USA, and Iran) producing 43% (FAO, 2017). Sweet cherry is greatly valued by consumers due to its taste, color, nutritional value, and beneficial health effects. Important quality characteristics of cherry fruits are weight, color, firmness, sweetness, sourness, flavor, and aroma (Crisosto et al., 2006). Sweet cherry is a highly perishable fruit containing significant levels of important nutrients such as potassium, dietary fiber, ascorbic acid, carotenoids, anthocyanins, and phenolic acids with only low caloric content (USDA, 2017). The objective of this review is to provide understanding of the cherry pre-and postharvest changes, and packaging technologies with regard to critical quality indicators and health benefits, thereby updating previous reviews by Predieri et al. (2003), Romano et al. (2006), McCune et al. (2010), and Wani et al. (2014). It also provides a peek on the road forwards with regard to the availability of new technologies. Part of these technologies are related to improving the circumstances in the chain as to gain access to long distance markets. Examples of these are the use of natural compounds and biodegradable films for modified atmosphere packaging (MAP) and edible coatings. The other part is related to improving the properties of the sweet cherry fruit itself; these are presented by showing the recent advances in marker assisted breeding preservation.

## Sweet cherry quality factors

### Maturity

Fruit maturity is one of the key factors determining overall fruit quality. Cherries should be harvested at the end of the maturation stage when they are fully ripe to ensure a good eating quality (Serrano et al., 2009). Although, the harvest timing varies based on the sweet cherry cultivars, there is harvest window of <5 days for harvesting fruit of optimal quality. The harvest season for sweet cherries is short and labor intensive. Ripe fruit are prone to mechanical damage and cooling directly after harvest is important (Dever et al., 1996; Chauvin et al., 2009).

During early developmental stages, skin color changes, and softening starts, glucose, and fructose accumulate, together with a rapid increase in fruit size. In later development stages, ascorbic acid and anthocyanin accumulate, antioxidant activity increases, and fruit flesh darkens (Serrano et al., 2005). A significant increase of total phenols was obtained in “Pico Negro” cherries during ripening and consequently, an increase in antioxidant activity, which may have beneficial health effects (Serradilla et al., 2012). Early-harvested cherries suffered from low acceptance due to the low sweetness while late-harvested cherries showed low acceptance due to low texture (Chauvin et al., 2009). Nevertheless, higher sensory scores were obtained for late-harvested sweet cherry fruits, especially for skin color and flavor intensity (Chauvin et al., 2009; Serradilla et al., 2012). Harvested cherries show weight loss, changes in the sugar-acid balance, color, softening, and stem browning (Kappel et al., 2002; Bernalte et al., 2003; Alique et al., 2005). During postharvest storage (4 days at 20°C) “Ambrunés” fruits showed a decrease in sugar levels, skin color, firmness, and TA, whereas SSC remained fairly stable, decreasing from 15.2 to 14.8°Brix. Malic acid levels decreased up to 20%, which may indicate that malic acid is an essential substrate for respiration in cherries (Alique et al., 2005).

Assessment of the optimal maturity in the orchard is not straightforward due to the variation in maturity within a tree, but especially within an orchard. Orchard mapping can support growers finding the correct harvest window by sampling°Brix and firmness during growth, just as now is being applied for dry matter assessment per plot for mango (Subedi et al., 2013). This type of quality mapping (Zude-Sasse et al., 2016) may be helpful to scheduling future cherry harvest campaigns.

### Color, firmness, and water loss

Cherry fruit quality traits related to consumer purchase decisions are based on external quality attributes such as color, fruit size, stem freshness, absence of defects, and stem length (Predieri et al., 2004). Aroma, flavor, sourness, sweetness, and texture are also essential attributes (Romano et al., 2006; Díaz-Mula et al., 2009). Fruit color is, however, the main quality trait (Mozetič et al., 2004). Color development has been studied as function of cultivar, ripeness stage, and storage conditions (Esti et al., 2002; Gonçalves et al., 2007; Pérez-Sánchez et al., 2010). Large variation exists amongst sweet cherry cultivars grown in Italy with the skin color variation classified as dark—(e.g., “Black Star” and “Moreau”) and light type cultivars (e.g., “Gabbaladri” and “Napoleona Verifica”; Ballistreri et al., 2013). Dark red blush cultivars have a higher consumer preference when comparing with full bright red cherries (Pérez-Sánchez et al., 2010). Color change during maturation is mostly due to an increase in anthocyanin content, specifically, cyanidin-3-*O*-rutinoside and cyanidin-3-*O*-glucoside (Mozetič et al., 2004; Gonçalves et al., 2007; Serrano et al., 2009). Transcriptomic studies in cherry fruit maturation have been carried out to elucidate the role of abscisic acid (ABA) and ethylene (Ren et al., 2011) and ripening (Luo et al., 2014). *PacNCED1* transcription induced ABA synthesis and accumulates during cherry ripening, which should play a crucial factor in regulation on the sweet cherry fruit ripening (Ren et al., 2011; Luo et al., 2014). The ethylene production via regulation of *PacACO1* expression (1-aminocyclopropane-1-carboxylic acid oxidase) might be stimulated with exogenous ABA, which indicates that the ethylene is synthesized by cherries (Ren et al., 2011) to start anthocyanin biosynthesis (Luo et al., 2014).

Positive effects of light on biosynthesis of phenolic compounds, mainly flavonoids are reviewed by Zoratti et al. (2014). Extensive studies have been carried out on the effect of supplemental UV-light treatments to improve anthocyanin levels in sweet cherry skin (Arakawa, 1993; Kataoka et al., 1996). Moreover, the application of UV-C light in postharvest has also been mentioned as a promise tool for extension of fruit shelf life, by delaying fruit senescence, increasing flavonoid content, and antioxidant activity in fruits (Wang et al., 2009; Crupi et al., 2013; Rivera-Pastrana et al., 2013; Li, 2014). The application of blue light emitting diodes (LEDs) to increase anthocyanin levels in cherries could be a reality soon (Arakawa et al., 2017), increasing both color and health promoting potential.

In general, consumers prefer cherries fully ripe based on their dark skin color (Crisosto et al., 2003). However, consumers chose partially ripe over full ripe “Sweetheart” cherries (Chauvin et al., 2009) which indicates the importance of a number of less well understood attribute interactions in consumer acceptance. In general, the chromatic values at harvest are lower at full ripeness stage than at the partially ripe stage. During postharvest storage, a further reduction in lightness (L^*^) and hue angle (*h*°) values were observed which indicates that the cherries became darker (Gonçalves et al., 2007). Firmness is related to storability, providing resistance to fruit deterioration and mechanical injury (Esti et al., 2002). Late season cultivars are frequently firmer than early season cultivars (Usenik et al., 2005). Fruit firmness at harvest varies greatly, from 3.3 for “Moreau” to 27 N for “Minnulara” cherries (Ballistreri et al., 2013). Firmness at harvest of 17 sweet cherry cultivars varied between 2.56 and 4.71 N and was judged acceptable for 73–92% of the panelists (Hampson et al., 2014). Softening is due to an increased peroxidase (POD), polyphenoloxidase (PPO), pectinmethylesterase (PME), polygalacturonase (PG), and β-galactosidase (β-Gal) activities. The level of these enzymes increased around 2 to 2.5-fold during a 5-day storage period causing a damage of cell wall components and subsequent softening (Remón et al., 2003).

The fruit cuticle likely plays an important role with respect to postharvest performance. The sweet cherry fruit cuticle is composed of a cutin polymer matrix, which consists essentially of esterified hydroxy- and epoxyhydroxy fatty acids with a chain length of 16 or 18 C-atoms and embedded in cuticular and surface-deposited epicuticular waxes (Peschel et al., 2007). During cherry development, the levels of C16 and C18 monomers decreased (Peschel et al., 2007), but increased during cold storage (Belge et al., 2014). During early sweet cherry fruit development, the cessation of cuticular membrane deposition is due to downregulation of genes involved in cuticular membrane deposition, such as *PaATT1, PaCER1, PaGPAT4/8, PaLACS 1, PaLACS2, PaLCR, PaLipase, PaLTPG1, PaWINA*, and *PaWINB* (Alkio et al., 2012). The main function attributed to the fruit cuticle is limiting water permeability, susceptibility to infections, and physiological disorders such as fruit cracking. The fruit cuticle composition is also associated with postharvest water loss from fruit respiration and firmness changes (Lara et al., 2014). Cherries have a high respiration rate which leads to softening that is partly due to water loss. Dehydration during postharvest storage might induce *PacNCED1* transcription and the accumulation of ABA resulting in ethylene production and subsequent fruit senescence (Luo et al., 2014). Water loss may lead to an increased susceptibility to infections and mechanical injuries (Esti et al., 2002). During postharvest storage, in particular for longer periods, fungal spoilage can lead to considerable economic losses (Conte et al., 2009; Romanazzi et al., 2009). The development of off-flavors is promoted by bacteria and fungi due to ethanol and acetaldehyde synthesis (Esti et al., 2002).

### Taste

Cherry fruit taste attributes, sweetness, and sourness, are important for consumer acceptance. Sweetness can be expressed as soluble solids content (SSC), sourness as titratable acidity (TA) with the ratio (SSC/TA) regarded as overall taste attribute (Guyer et al., 1993; Crisosto et al., 2002). SSC values varied from 12.3 to 23.5°Brix (González-Gómez et al., 2009) and varied from 14.7 to 23.7°Brix for Spanish sweet cherry cultivars (Pérez-Sánchez et al., 2010). SSC values ranged from 18.1 to 19.3 and from 17.8 to 19.8°Brix for Brazilian and Chinese sweet cherries, respectively (Rios de Souza et al., 2014; Wen et al., 2014). TA values ranged from 0.20 in “Lapins” to 0.28 for the Canada Giant cultivars (Vavoura et al., 2015). High TA affects taste when the SSC value is below 16°Brix in “Brooks” or below 13°Brix in “Bing” (Crisosto et al., 2003). The optimal SSC/TA ratio is between 1.5 and 2.0, with SSC values ranging between 17 and 19°Brix (Kappel et al., 1996).

Sweetness and flavor intensity are important parameters for sweet cherry liking (Romano et al., 2006). The most important sensory features are related to the ripening stage, parameters linked with accumulation of organic acids and aromatic alcohols (Serradilla et al., 2012). Several pathways in the biosynthesis of volatiles originate from amino acids, membrane lipids, and carbohydrates contributing for the ripe fruit flavor (Hadi et al., 2013). The aroma profile of sweet cherries is mainly the result of a complex mixture of alcohols, carbonyls, and organic acids (Mattheis et al., 1992; Girard and Koop, 1998; Bernalte et al., 1999; Zhang et al., 2007; Serradilla et al., 2012; Vavoura et al., 2015). Hexanal and ethyl-2-hexenal are the most relevant compounds in the aroma profile of sweet cherries, that together with ethyl-2-hexen-1-ol, are associated with fresh green odors and green notes (Zhang et al., 2007; Serradilla et al., 2012). Benzaldehyde, originates from the enzymatic hydrolysis of amygdalin, is likely the main contributor to the characteristic flavor of sweet cherry (Zhang et al., 2007). Other important contributors are hexanoic acid, described as floral (Pherobase, 2007), and acetic acid, the main volatile acid found in sweet cherry fruits (Serradilla et al., 2012). Significant changes in volatile composition have been observed during ripening of sweet cherry (Zhang et al., 2007; Serradilla et al., 2012). The branched-chain alcohols, 3-methyl-3-buten-1-ol, and 3-methyl-2-buten-1-ol increase during ripening, whereas the organic acids, tetradecanoic and hexadecanoic acid, decrease during ripening, which may be due to reactions between alcohols and organic acids, catalyzed by alcohol acyltransferases. These enzymes seem to play a crucial role in ester biosynthesis (Olías et al., 1995; Serradilla et al., 2012). High variation was recorded in the composition of volatiles amongst four sweet cherry cultivars grown in Spain (Serradilla et al., 2012). “Sweetheart” cherries were mainly characterized by higher levels of organic acids, such as 9-hexadecenoic acid, hexadecanoic acid, and aromatic alcohols and “Ambrunés” cherries by higher sweetness levels and presence of aliphatic alcohols. “Pico Colorado” cherries showed higher levels of hexanal and “Pico Negro” cherries were characterized by higher levels of branched alcohols, mainly 3-methyl-2-buten-1-ol and aromatic aldehydes, such as benzaldehyde. Similar research with four sweet cherry cultivars grown in Greece reported that “Ferrovia” and “Skeena” cherries showed higher levels of carbonyl compounds (2-propanone and 2-hexenal) and alcohols compared to “Lapins” and “Canada” cherries (Vavoura et al., 2015).

## Health promoting compounds

The role of several classes of health promoting compounds that are present in sweet cherry, lowering the risk of cancer, cardiovascular disease, diabetes, and other inflammatory diseases, has been recognized and is a field of many animal and now increasingly also epidemiological studies (McCune et al., 2010).

### Phenolic compounds

Sweet cherry phenolic compounds exhibit high antioxidant activity (Gonçalves et al., 2004a; Serrano et al., 2005; Kelebek and Selli, 2011; Serra et al., 2011). These compounds consist of flavonoids, flavan-3-ols, and flavonols and non-flavonoid compounds like hydroxycinnamic and hydroxybenzoic acids (Macheix et al., 1990; Gao and Mazza, 1995; Gonçalves et al., 2004a). Phenolic compounds, concentrated in the fruit skin, increase during ripening together with polyphenolic compounds and anthocyanins that color the cherry skin from green to red (Mozetič et al., 2004; Gonçalves et al., 2007). Anthocyanins and flavonols contribute to sensory and organoleptic properties of fruits (Ferretti et al., 2010). Table 1 presents an overview of the levels of phenolic compounds found in several sweet cherry cultivars. The most abundant phenolic compounds are anthocyanins (Mozetič et al., 2002; Gonçalves et al., 2004a; Usenik et al., 2008) such as cyanidin-3-*O*-rutinoside and cyanidin-3-*O*-glucoside, peonidin-3-*O*-rutinoside and glucoside, as well as pelargonidin-3-*O*-rutinoside (Gonçalves et al., 2007). Anthocyanins have health promoting properties. For instance, cyanidin-3-*O*-rutinoside slows down absorption of carbohydrates which may help prevent or treat diabetes mellitus (Adisakwattana et al., 2011) and cyanidin-3-*O*-glucoside showed cardio-protective effects by reducing blood lipid levels (Xia et al., 2005). Several studies reported higher phenolic content (Gonçalves et al., 2004a; Serrano et al., 2009), SSC, TA and antioxidant activity (Serrano et al., 2009) in ripe cherries than in partially ripe. Sweet cherry contains phenolic acids such as hydroxycinnamic acid derivatives (neochlorogenic, *p*-coumaroylquinic, and chlorogenic acids; Gonçalves et al., 2004a; Usenik et al., 2010; Liu et al., 2011), flavonols (quercetin-3-glucoside, quercetin-3-rutinoside, and kaempferol-3-rutinoside) and flavan-3-ols (catechin and epicatechin), as shown in Table 1 (Gonçalves et al., 2004a; Mozetič et al., 2006; Usenik et al., 2008; Jakobek et al., 2009). Consumption of cherries might induce health benefits such as inhibition of tumor growth (Kang et al., 2003; Serra et al., 2011), inhibition of inflammation (Seeram et al., 2001; Jacob et al., 2003) and protection against neurodegenerative diseases (Kim et al., 2005). These fruits are also considered an excellent source of polyphenols, such as tannins (Tomás-Barberán and Espín, 2001). Tannins can be classified in hydrolysable tannins (gallic acid polymerization) and condensed tannins or proanthocyanidins (catechin polymerization) (Macheix et al., 1990). Tannins are also secondary metabolites that confer astringency and have health-promoting properties (Tomás-Barberán and Espín, 2001; McCune et al., 2010). Tannin content ranged from 32 to 75 mg 100 g^−1^ of fresh weigh for Serbian sweet cherry cultivars (Prvulović et al., 2012). Sweet cherry extracts might be a serious candidate to prevent oxidative stress-induced disorders like intestinal inflammation disorders and neuronal cell death (Matias et al., 2016).

**Table 1:** Content of phenolic compounds (mg 100 g^−1^ FW) in different sweet cherry cultivars.

**Cultivar**	**Hydroxycinnamic acids**			**Flavan-3-ols**		**Flavonols**	**Anthocyanins**					**References**
	**NcAc**	***p*CqAC**	**CAc**	**Cat**	**Epi**	**Rut**	**cy-3-glu**	**cy-3-rut**	**pn-3-glu**	**plg-3-rut**	**pn-3-rut**	
Ferrovia	–	–	–	–	–	–	3.0	60.3	<1.0	<1.0	<1.0	Esti et al., 2002
Sciazza	–	–	–	–	–	–	48.0	393.0	3.0	3.5	27.5	
Burlat	23.8	24.7	3.8	7.2	6.7	4.8	23.2	44.6	<1.0	<1.0	2.1	Gonçalves et al., 2004a
Saco	153.5	12.2	9.8	10.5	10.3	11.8	5.1	38.6	n.d.	<1.0	<1.0	
Summit	34.4	27.5	7.2	5.8	8.2	3.1	2.4	26.0	<1.0	<1.0	<1.0	
Van	65.6	5.6	4.8	3.5	4.5	4.0	3.4	28.2	<1.0	<1.0	1.5	
Burlat	6.8	6.4	1.1	–	3.1	4.5	2.3	8.3	–	<1.0	<1.0	Usenik et al., 2008
Lapins	8.7	<1.0	1.7	–	<1.0	2.1	<1.0	3.1	–	<1.0	<1.0	
Sylvia	7.3	7.2	<1.0	–	2.9	3.7	<1.0	9.8	–	<1.0	<1.0	
Van	17.3	4.2	5.8	4.5	6.3	3.6	1.5	43.6	0.6	<1.0	1.2	Kelebek and Selli, 2011
Burlat	64.2	–	4.2	–	9.7	1.8	7.1	44.5	n.d.	n.d.	n.d.	Liu et al., 2011
Colt	5.5	–	6.7	–	<1.0	2.6	<1.0	12.3	n.d.	n.d.	n.d.	
Lapins	64.6	–	4.3	–	2.5	1.6	<1.0	21.7	n.d.	n.d.	n.d.	
Lapins^*^	85.5	6.1	8.9	8.6	10.6	23.4	70.3	162.0	4.5		5.4	Serra et al., 2011
Saco^*^	123.0	15.2	8.7	7.5	9.4	26.6	55.6	282.0	5.3		3.5	
Van^*^	61.9	4.6	5.3	7.4	2.6	34.2	70.1	253.0	1.8		2.5	
Burlat	9.2	11.3	1.8	–	–	–	34.8	46.9	–	<1.0	2.3	Ballistreri et al., 2013
Sweetheart	9.9	6.4	1.5	–	–	–	1.39	22.4	–	<1.0	3.8	

NcAc, Neochlorogenic acid; pCqAC, p-Coumaroylquinic acid; CAc, Chlorogenic acid; Cat, Catechin; Epi, Epicatechin; Rut, Rutine; cy-3-glu, Cyanidin-3-O-glucoside; cy-3-rut, Cyanidin-3-O-rutinoside; pn-3-glu, Peonidin-3-O-glucoside; plg-3-rut, Pelargonidin-3-O-rutinoside; pn-3-rut, Peonidin-3-O-rutinoside; n.d., not detected; not determined; FW, Fresh weight; DW, Dry weight;

* *(mg 100 g^−1^ DW)*.

### Vitamins and carotenoids

Cherries are rich in C, A, E, and K vitamins and carotenoids, specially β-carotene, lutein, and zeaxantine (Ferretti et al., 2010; Leong and Oey, 2012). Ascorbic acid (vitamin C) levels vary between 4 and 7 g kg^−1^ of fresh weight among seven sweet cherry cultivars grown in Turkey (Demir, 2013), whereas the ascorbate levels for 22 sweet cherry cultivars grown in southern of Italy was considerably lower, varying from 0.034 to 0.260 g kg^−1^ of fresh weight (Matteo et al., 2016). Carotenoids, such as β-carotene, β-cryptoxanthin, and α-carotene have been reported in sweet cherry fruit (Leong and Oey, 2012; Demir, 2013; Matteo et al., 2016). These compounds are the main precursors of vitamin A and responsible for the red, yellow and orange hues in the cherry skin. β-carotene, β-cryptoxanthin and α-carotene, lycopene, and lutein are present in cherry at levels of 0.02 mg g^−1^ dry weight (Leong and Oey, 2012). β-carotene, β-cryptoxanthin, and α-carotene, zeaxantine were identified in seven sweet cherry cultivars in small quantities varying from 0.2 to 16 ppm of fresh weight (Demir, 2013). Cherry carotenoids levels are generally quite low, and likely do not present a large contribution to human health.

Nowadays an increasing trend in society is health consumerism, for example functional drinks with cherry extracts rich in bioactive compounds (Sun-Waterhouse, 2011). However, it is important to evaluate the impact of natural ingredients on sensory properties and consumer acceptance, as the optimal combination of taste and health promoting compounds is difficult to achieve (Corbo et al., 2014).

## How do growth conditions affect fruit quality and the level of health promoting compounds?

### Scion × rootstock interaction

Several studies have shown the influence of the scion × rootstock combination on cherry fruit quality (Schmitt et al., 1989; Facteau et al., 1996; Shackel et al., 1997; Szot and Meland, 2001; Whiting et al., 2005). Fruit firmness varies with scion x-rootstock combination. For instance, “Burlat” cherries are softer when grafted on CAB 11E (clone of *Prunus cerasus* L.; semi-vigorous) and firmer when grafted on Gisela 5 (*P. cerasus* × *P runus canescens* Bois. hybrid; dwarfing) or *P. avium* (Gonçalves et al., 2006). Fruits grown on PiKu 1 and Weiroot 13 rootstocks had high levels of chlorogenic acid, neochlorogenic acid, *p*-coumaric acid and quercetin-3-rutinoside compared to F12/1, Gisela 5, Maxma 14, and Weiroot 158 rootstocks (Jakobek et al., 2009). The lowest stone weight (7% of total fruit weight) and highest level of total sugars (250 g kg^−1^ fresh weight) was observed for “Lapins” on F12/1 rootstock, whereas PiKu 1 rootstock resulted in the highest yield (20 kg tree^−1^). The Weiroot 13 rootstock promoted the highest content of total organic acids (9 g kg^−1^ of fresh weight) and the Edabriz rootstock resulted in the highest SSC (15°Brix). “Lapins” grown on the Weiroot 72 rootstock presented higher fruit weight, firmness, SSC, and phenols content (Usenik et al., 2010). Choosing the right rootstock continues to be widely studied due to its importance for productivity (Koc et al., 2013) but the effects on fruit quality are also of increasing interest (Milinović et al., 2016).

### Orchard management

Nowadays, the big challenge in cherry production is to reduce the costs and the labor requirements. This implies the use of the right rootstock and the best canopy architecture. Mechanization of pruning and a fully mechanical harvest system will facilitate achieving higher yield and fruit quality (Lang, 2017).

Summer pruning, which can provide adequate vigor and good light penetration throughout the canopy, and a high leaf area per fruit are important factors for the production of high quality fruits (Whiting and Lang, 2004). Tree location in the orchard and fruit position in the tree are the most important factors that affect fruit weight (Drake and Fellman, 1987; Flore and Layne, 1999). Fruit size and sweetness decrease with increasing plant density, which indicates competition between trees (Eccher and Granelli, 2006). High light intensity increases ascorbic acid content, and high temperature enhances anthocyanin and total phenolic contents (Wang, 2006).

Canopy architectures, such as Y-trellised and vertical, Tall Spindle Axe, Kim Green Bush, Super Slender Axe, and Upright Fruiting Offshoots, have recently been implemented in several orchards (Lang et al., 2017) with the aim to improve the crop loading, the light input, as well as, the increase of health relating compounds content in cherries.

Especially in Mediterranean areas, due to water scarcity, improvement of cherry production under water stress is essential. Several studies evaluated irrigation systems with regard to productivity and fruit quality (Demirtas et al., 2008; Yin et al., 2011; Neilsen et al., 2014). Drip irrigation applied to “Lapins” cherry trees consumed only 21–29% of the irrigation water compared to micro-sprinkler irrigation, enhanced marketable fruit by 7 to 12% and did not impact fruit yield or firmness, color, and size (Yin et al., 2011). Low-frequency drip irrigation increased SSC in “Cristalina” and “Skeena” cherries (Neilsen et al., 2014). However, irrigation levels did not affect SSC, pH, TA, and fruit weight of the cherries of the 900-Ziraat cultivar (Demirtas et al., 2008). Water management techniques, such as regulated deficit irrigation (RDI) and partial rootzone drying (PRD) may enhance fruit taste without reduction of yield (Ripoll et al., 2016). Applying RDI, to “Summit” sweet cherry trees after the crop was harvested did not impact yield but could save water up to 45% without affecting firmness and limiting thinning costs (Marsal et al., 2010). Limiting water supply during cherry tree development, either by drip irrigation or RDI, may help to produce healthier food a more sustainable manner (Nora et al., 2012), and would especially be important taking into account the effects of climate change.

Cherry production depends on the pollinators and utilization of pollinizer cultivar in the orchard. However, in a near future, these can be replaced by artificial precision pollination systems using liquid pollen suspensions applied with electrostatic sprayer technology (Whiting and Das, 2017).

### Growth regulators

The application of calcium and growth regulators improves cherry quality (Table 2). Application of Ca(OH)_2_ 0.7% reduced cracking and fruit size and increased SSC, firmness, and calcium levels in both fruit flesh and skin (Demirsoy and Bilgener, 1998a). Sweet cherry trees sprayed with CaCl_2_ 0.5% once a week from petal fall until 2 weeks before harvest showed higher SSC and higher levels of phenolics, reduced decay, and cuticular fractures, but showed higher weight loss during storage (Vangdal et al., 2008). Preharvest application of ethephon reduced both fruit removal force and fruit flesh firmness at harvest (Elfving et al., 2009). Fruits treated with 20 ppm gibberellic acid (GA_3_) at the green to straw-yellow stage showed slower softening with lower PG and cellulase activity, but no effects on SSC and β-Gal activity (Choi et al., 2002). GA_3_ can induce, depending on the cultivar, increased fruit weight and SSC, including delayed ripening and reduced fruit cracking (Usenik et al., 2005). GA_3_ application increased fruit firmness of “Lapins” and “135-27-17”cherries, but no effect was observed in “Celeste” and “Merpet” fruits (Choi et al., 2002). Similar preharvest applications of GA_3_ (25 ppm) at the straw-yellow stage of the “0900 Ziraat” cherries showed increased fruit size and firmness (Canli and Orhan, 2009). Applications of GA_3_ (30 and 60 mg L^−1^) to cherries at the straw-yellow stage of the Regina, Sweetheart and 0900 Ziraat cultivars retarded fruit ripening and preserved flesh firmness. However, these fruits showed a decrease in total phenolics, anthocyanins, and antioxidant activity (Ozkan et al., 2016). Preharvest application of 25, 50, and 100 mg L^−1^ GA_3_ and GA_3_ combined with 0.05, 0.1, and 0.5 mg L^−1^ 22S, 23S-homobrassinolide (HBR) to “Regina” and “Summit” cherries at full bloom and at the beginning of fruit development increased fruit size and decreased SSC (Engin et al., 2016). Fruits treated with only HBR showed higher fruit firmness of the flesh and stem resistance (Engin et al., 2016). At the straw-yellow stage applications of “Noir de Guben” cherries with 50, 100, and 150 ppm benzyladenine (BA) and BA combined with 12.5, 25, and 50 ppm gibberellins (GA_4+7_) improved size and delayed skin color change (Canli et al., 2015a). BA alone or in combination with GA_4+7_ improved storability by maintaining firmness during 30 days in cold storage (4°C) (Canli et al., 2015b). GA_3_ applied at stage I (mesocarp growth consisting of both cell division and elongation) and stage II (endocarp hardening and embryo development) improved final “Bing” fruit weight, but higher fruit weight was also obtained when combined with GA_4+7_ (Zhang and Whiting, 2011). Application of salicylic acid (SA) and acetylsalicylic acid (ASA) at 0.5 and 1 mM at three key points of fruit development improved fruit weight up to 59%, increased firmness and increased anthocyanin and total carotenoid content (Giménez et al., 2014). Application of 2 mM oxalic acid (OA) increased fruit size up to 23%, increased levels of anthocyanins, flavonols, neochlorogenic, and chlorogenic acids (Martínez-Esplá et al., 2014). The growth regulators application improves final fruit size due to the stimulating cell division (Olmstead et al., 2007). However, the successful application of these compounds is highly dependent on climatic conditions and time of application. Methyl salicylate (MeSA) application increased fruit size, red coloring, firmness, and SSC (Giménez et al., 2015). MeSA also increased total phenolics, anthocyanins, and induced higher activity of antioxidant enzymes such as ascorbate peroxidase, catalase, peroxidase, and superoxide dismutase at harvest (Valverde et al., 2015). Interestingly, MeSA coatings affect postharvest quality of cherry fruits while still attached on the tree. Total phenolic and anthocyanin contents, total antioxidant activity and antioxidant enzymes were higher in MeSA-treated fruits during storage (Valverde et al., 2015), while also delaying postharvest ripening due to less coloring, lower acidity, and less softening in treated fruits after storage (Giménez et al., 2015). Application of preharvest coatings may be an exciting new trend in research that could be instrumental to enable further access to long distance markets as both storability and the levels of health promoting compounds are improved. In Portugal, the cherry growers are currently applying biostimulants based on seaweed and tertiary ammonium compounds (such as glycine betaine), and it is likely that, observing the positive (unpublished) results that have been obtained, that application of these compounds might be common in the near future.

**Table 2:** Effect of calcium and growth regulators on quality attributes of several sweet cherry cultivars.

**Source/Compound**	**Cultivar**	**Effects**	**References**
Ca(OH)_2_	0900 Ziraat Lambert Van	Higher firmness, SSC and calcium content both in skin and flesh; Reduced fruit cracking; Decreased fruit size.	Demirsoy and Bilgener, 1998a
CaCl_2_	Merton Glory Sue Vega	Higher SSC and phenolics content; Reduced decay and cuticular fractures; Non-significant effects on TA, color and firmness; Increased weight loss during storage.	Vangdal et al., 2008
Ethephon	Bing	Higher firmness (dehydration-related); Reduced fruit removal force and firmness.	Elfving et al., 2009
Gibberellic acid (GA_3_)	Buttner's Red Lambert Van	Suppress or delay the development of the pitting symptom on bruised fruit.	Looney and Lidster, 1980; Basak et al., 1998
	0900 Ziraat Bing Lambert	Delayed harvest time.	Facteau et al., 1985; Andrews and Shulin, 1995; Demirsoy and Bilgener, 2000
	Bing Satohnishiki	Decreased the activity of PG and PME.	Andrews and Shulin, 1995; Kondo and Danjo, 2001
	Buttner's Red	Increased fruit firmness, SSC and fruit weight.	Basak et al., 1998
	0900 Ziraat Lambert Van	No significant effect on fruit firmness, SSC and fruit weight.	Demirsoy and Bilgener, 1998b
	13S-27-17 Celeste Lapins Merpet	Increased fruit firmness and TA; Decreased the activity of PG and cellulase activity; Delayed softening and fruit maturation; No significant effect on SSC and β-glucosidase activity.	Choi et al., 2002
	Bing	Higher firmness during storage.	Clayton et al., 2003
	Sweetheart	Firmer, heavier and larger fruits, better preservation of stem; No effect on color and SSC content.	Horvitz et al., 2003
	Elisa Sunburst Van	Higher yield; Increased fruit weight and SSC; Delayed process of maturity; Reduced fruit cracking.	Usenik et al., 2005
	0900 Ziraat	Firmer and larger fruits; Higher SSC.	Canli and Orhan, 2009
	Bing	Increased fruit firmness and weight; Delayed fruit maturation; Decreased fruit color.	Zhang and Whiting, 2011
	0900 Ziraat Regina Sweetheart	Increased fruit size; Retarded fruit ripening; Preserved flesh firmness; Decreased SSC, total phenolics, anthocyanins accumulation and antioxidant activity.	Ozkan et al., 2016
22S, 23S-Homobrassinolide (HBR)	Regina Summit	Increased fruit size and brightness of the red color.	Engin et al., 2016
		Increased fruit size and firmness of the flesh slightly; Better stem resistance.	
GA_3_ + HBR		Increased fruit size; Higher longer fruit; Decreased SSC.	
Benzyladenine (BA) + Gibberellin (4+7)	Noir de Guben	Increased fruit size; Delayed skin color development; No significant effect on fruit firmness.	Canli et al., 2015a
Cytokinins		Increased fruit firmness, SSC and fruit weight; Decreased fruit color.	Zhang and Whiting, 2011
Auxins	Bing	Increased fruit size and total yield.	Stern et al., 2007
		Higher fruit growth rates and fruit color development (Stages I and II); No significant effect on fruit size.	Zhang and Whiting, 2011
Methyl jasmonate (MeJA)	n.d.	Reduced incidence of postharvest rot; Decreased β-1,3-glucanase, PAL and POD activities.	Yao and Tian, 2005
Salicylic acid (SA)	Sweetheart Sweet Late	Increased fruit firmness and weight; Higher concentration in total phenolics and anthocyanins; No effect on SSC and TA.	Giménez et al., 2014
Acetylsalicylic acid (ASA)		Increased fruit firmness and weight; Higher concentration of total phenolics and anthocyanins; No effect on SSC and TA.	
Oxalic acid (OA)		Increased fruit size at harvest, color and firmness; Higher concentration of total phenolics and anthocyanins; No significant effect on SSC and TA.	Martínez-Esplá et al., 2014
Methyl salicylate (MeSA)	Lapins Sweetheart Sweet Late	Increased fruit size, firmness and SSC; Higher activities of total antioxidant activity and antioxidant enzymes; Higher total phenolics and anthocyanins content; Delayed the postharvest ripening process.	Giménez et al., 2015; Valverde et al., 2015

*SSC, soluble solids content; TA, titratable acidity; PAL, phenylalanine ammonia-lyase; POD, peroxidase; PG, polygalacturonase; PME, pectinmethylesterase; n.d., not defined*.

## How to maintain shelf-life and health promoting compounds in the chain?

### Storage conditions

Optimization of storage and transport conditions are important tools to extend and preserve shelf life and thus allow long-distance transport to destination markets (Remón et al., 2003; Martínez-Romero et al., 2006). Temperature and relative humidity (RH) are critical factors that influence cherry quality during postharvest storage (Kader, 2001; Romano et al., 2006). According to Bernalte et al. (1999), the optimal temperature to store sweet cherries is 0.5°C at 90% RH. During cold storage, the level of phenolic compounds such as neochlorogenic acid, *p*-coumaroylquinic acid, chlorogenic acid, rutin, catechin and epicatechin, and the content of anthocyanins such as cyanidin-3-*O*-rutinoside and peonidin-3-*O*-rutinoside increased in several sweet cherry cultivars (Gonçalves et al., 2004a,b, 2007; Serrano et al., 2009). The phenolics content in “Canada Giant” and “Ferrovia” cherries increased during at storage at 20°C during 8 days (Goulas et al., 2015), probably due to the condensation of the phenolic compounds by water loss during ripening or by postharvest synthesis (Kalt et al., 1999). However, Esti et al. (2002) observed a decrease of 41–52% of total anthocyanins content after 15 days at 1°C and 95% RH in two sweet cherry cultivars, which may indicate no net anthocyanin biosynthesis.

Hydro-cooling reduced sweet cherry stem browning and surface shriveling, while decay, external color, and SSC were not affected in “Tragana Edessis” and “Mpakirtzeika” cherries after 1-week of cold storage (0°C, 95% RH) (Manganaris et al., 2007). Adding CaCl_2_ during hydro-cooling reduced the respiration rate, ascorbic acid degradation, and membrane lipid peroxidation. Moreover, total phenolic content, antioxidant capacity, and fruit firmness increased and TA slightly decreased in “Sweetheart” and “Lapins” cherries (Wang Y. et al., 2014). Hydro-cooling can effectively increase quality of cherries by the reduction in temperature of the cherries after harvest, the sooner the better. Hydro-cooling portable equipment that can be installed in the orchard might be a good option (Elansari, 2009).

In order to extend the self-life of sweet cherries, MAP, and controlled atmosphere (CA) storage have been successfully implemented. Optimum CO_2_ and O_2_ levels for long time storage have been established at 2–10% for O_2_ and 5–20% for CO_2_ (Meheriuk et al., 1997; Remón et al., 2000, 2003; Kupferman and Sanderson, 2001; Tian et al., 2001; Spotts et al., 2002). Lower O_2_ levels may result in a decrease of volatile aromatic compounds and development of off-flavors (Meheriuk et al., 1995; Remón et al., 2000; Kader, 2002), while CO_2_ levels higher than 30% have been related to a brown discoloration of the skin (Kader, 1997). High O_2_ levels (70% O_2_ + 0% CO_2_) can inhibited ethanol production, but also induced a rapid decrease in ascorbic acid content and often resulted in browning due to high-O_2_ injury (Tian et al., 2004). Super atmospheric O_2_ packaging (100 kPa O_2_), in polypropylene film during 10 days at 4°C and 70% RH delayed respiration rate of cherries, inhibited ethylene production and PPO and POD activities and maintained firmness, soluble proteins and SSC for the first 4 days of storage. However, ascorbic acid content was lower compared to control cherries (Wang S. et al., 2014). MAP combined with low temperature storage reduces the incidence of fruit decay while preserving SSC, TA, and stem color. Fruits also displayed higher antioxidant capacity, firmness and, a brighter colored skin (Alique et al., 2003; Remón et al., 2003; Padilla-Zakour et al., 2004; Lara et al., 2015). High CO_2_ levels and cold storage both favor net anthocyanin synthesis as anthocyanin biosynthesis is induced during the postharvest cold storage of several red fruits (Remón et al., 2000; Conte et al., 2009). This increase may be due to inhibition of PPO activity promoted by CO_2_-enriched atmospheres, since changes in fruit color occurred due to oxidation of phenols by PPO (Vamos-Vigyazó, 1981). MAP and CA stored sweet cherry fruits at 5% O_2_ combined with 10% CO_2_ at 1°C reduced PPO and POD activity, prevented flesh browning, decreased fruit decay and promoted firmness retention. Weight loss, pitting, stem visual appearance, and PG and PPO activity, of “0900 Ziraat” cherries were lower when applying modified atmosphere-modified humidity packaging (MA/MH) using 0.20 μm low-density polyethylene during 8 days at 0°C and a 2-day shelf-life (Özkaya et al., 2015). Certainly, MAP can be useful for sweet cherry shelf-life extension, but large scale implementation in practice is limited as it is expensive and somewhat risky due to possible gas mixture mismanagement and fungal growth.

## New frontiers for sweet cherry quality improvements

### Natural compounds

Natural compounds have been applied to improve the shelf life of cherries. Application of 1 mM SA or ASA or OA on cherries stored for 20 days at 2°C showed lower acidity, less color changes and firmness loss and maintained higher levels of health promoting compounds and antioxidant activity (Valero et al., 2011). Immersion in 30 mM β-aminobutyric acid (BABA) for 10 min reduced respiration rate, weight loss and increased TA levels, total phenolics, sugars and ascorbic acid of “Hongdeng” cherries during 5 days at 20°C. Moreover, BABA-treated cherries showed higher ascorbate peroxidase, catalase, glutathione reductase, and superoxide dismutase activities (Wang et al., 2016). The use of these compounds is considered safe and environmentally friendly, resulting in extending storability of sweet cherry with increased health-promoting properties. Currently, these postharvest treatments have only been applied at the research level, but the future is promising for application in practice.

### Biodegradable films for MAP and edible coatings

Preservation technologies have emerged using biodegradable films for MAP (Giacalone and Chiabrando, 2013; Koutsimanis et al., 2014a,b). Biodegradable films have a reduced environmental footprint (Almenar et al., 2012) and delay color changes, softening and loss of acidity (Giacalone and Chiabrando, 2013). Micro-perforated polylactic acid (PLA) film inhibited fungal growth, maintained brightness, skin color, firmness, SSC, and decreased fruit weight loss in sweet cherry when compared with macro-perforated bags. Cherries stored in PLA packages showed better appearance, texture, flavor, and overall acceptability (Koutsimanis et al., 2014a). This packaging technology may become practice (Koutsimanis et al., 2014b), but depends on the balance between (less) waste production and (higher) price for these type of films (Tharanathan, 2003; Vroman and Tighzert, 2009).

Edible coating is a promising technology in the preservation of fruits and vegetables quality (Dhall, 2013). Edible fruit coatings can also provide an alternative to modified atmosphere storage and act as carriers of health promoting compounds. Bioactive compounds, such as antioxidants, antimicrobials, probiotics, flavors, and nutraceutical substances, can be transported from the coating onto the fruit skin by diffusion, enhancing shelf life, and nutritional quality (Quirós-Sauceda et al., 2014). Edible coatings may reduce respiration and transpiration rates, reduce firmness loss, and limit decay (Velickova et al., 2013). Locust bean gum, shellac, polysorbate 80, glycerol, and beeswax coatings applied to “Burlat” cherries showed lower weight loss, less bruising injury, less surface pitting and lower flesh firmness loss resulting in a prolonged shelf-life (Rojas-Argudo et al., 2005). Chitosan is an antimicrobial compound that induces fungal defense-related enzymes and enhances the level of phenolic compounds (Liu et al., 2007). Chitosan-coated sweet cherries showed lower water loss, delayed color changes, higher total phenolics, flavonoid, and anthocyanin levels (Petriccione et al., 2014). Misir et al. (2014) reviewed the application of *aloe vera* gels as an edible coating for fresh fruits to enhance the postharvest life and quality attributes of, amongst others, sweet cherries. *Aloe vera* gel reduced weight loss and lowered the respiration rate, delayed color changes, softening, and TA losses in cherry fruit compared to control fruits (Martínez-Romero et al., 2006). According to the European Directive (ED European and Parliament Council Directive N 95/2/EC, 1995; ED European Parliament and Council Directive N 98/72/EC, 1998) and USA regulations (FDA, 2016), edible coatings can be classified as food products, food ingredients or food additives. This allows for application of edible coatings in practice, although more studies are needed in order to improve functionality and consumer acceptance.

### Marker assisted breeding

The recent publication of the huge and complex sweet cherry genome (Shirasawa et al., 2017) will allow for identification of candidate genes, signal transduction-, and metabolic pathways, which can have a relevant role in fruit quality (Carrasco et al., 2013). Sweet cherry linkage maps have been constructed to facilitate identification and characterization of quantitative trait loci (QTLs) associated with quality traits (Olmstead et al., 2008; Guajardo et al., 2015). QTLs have been identified for fruit weight and firmness (Campoy et al., 2015), fruit size (Zhang et al., 2010; Franceschi et al., 2013; Rosyara et al., 2013), skin and flesh fruit color (Sooriyapathirana et al., 2010), and fruit cracking tolerance (Balbontín et al., 2013). About 133 genes are putatively involved in fruit texture, color, flavor, and chilling injury resistance (Ogundiwin et al., 2009). Moreover, in sweet cherry, exocarp-specific transcripts associated with epidermis development (from flowering to maturity) and stress responses (Alkio et al., 2014) were established. In stone fruit breeding programs, marker-assisted selection (MAS) is an important biotechnological tool, although its use is currently limited due to the polygenic nature of traits linked to fruit quality (Salazar et al., 2014). Genotyping by sequencing (GBS) generates more saturated genetic maps, thus facilitating the identification of (more) accurate QTLs and the discovery of single nucleotide polymorphisms (SNPs) (Guajardo et al., 2015; Salazar et al., 2017), with focus mainly on finding sweet cherry quality QTLs and not on finding QTLs that affect the level of health promoting compounds. The use of DNA information, as clustered regularly interspaced short palindromic repeats (CRISPR) along with CRISPR-associated protein 9 (Cas9) system (CRISPR/Cas9), appears to be relevant in understanding genetic variation, inheritance, genomic organization, and phenotypic performance, contributing to the development of new cultivars of Rosaceous crop breeding (Peace, 2017). Although, the CRISPR/Cas9 gene-editing technique has not yet been used in cherry, it has been effectively used to confer resistance against citrus canker (Peng et al., 2017), regulating ethylene synthesis (*Ripening inhibitor* gene) in tomato (Ito et al., 2015) and in the efficient knockout of the *L-idonate dehydrogenase* gene (*IdnDH*), involved in the tartaric acid pathway in grape (Ren et al., 2016).

Taking this into account, it is expected that CRISPR/CAS gene editing will be instrumental with regard to improving fruit quality and the level of health-related compounds.

## Concluding remarks and future perspectives

We are at the beginning of acquiring knowledge to understand the variation in health-related compounds throughout the fruit maturation and the postharvest phase. How environmental factors, dose and time of preharvest compounds application affects tree performance and consequently fruit quality is at the moment largely unknown. Further research should be aimed to develop cultivar-specific strategies that improves both fruit quality and fruit nutritional characteristics, without significantly affecting yield. Optimizing cherry quality attributes needs a better understanding of the interaction between genetics, environmental factors, and plant growth regulators to be able to gain full benefit of new preservation technologies. The incorporation of bioactive compounds may provide advantages in food preservation and contributes to the development of functional foods. Application of bioactive compounds and encapsulation with edible coatings are promising techniques that also need further investigation, also with regard to diminishing off flavors. New cultivars with improved characteristics such as fruit size and color, anthocyanin biosynthesis, and cracking resistance are needed in order to produce cultivars with exceptional quality, high consumer appeal, well adapted to local growing and future climatic conditions. With the recent advancements in marked assisted breeding, especially now that the draft sweet cherry genome has been published, exciting times await.

## Author contributions

SC did the literature research, drafted the manuscript and made tables. RS, AS, and BG helped writing the final manuscript and all authors approved the final manuscript.

### Conflict of interest statement

The authors declare that the research was conducted in the absence of any commercial or financial relationships that could be construed as a potential conflict of interest. The reviewer DO and handling Editor declared their shared affiliation.
