# Corrigendum: Factors Affecting Quality and Health Promoting Compounds during Growth and Postharvest Life of Sweet Cherry (*Prunus avium* L.)

**DOI:** 10.3389/fpls.2018.00464

**Published:** 2018-04-06

**Authors:** Sofia Correia, Rob E. Schouten, Ana P. Silva, Berta Gonçalves

**Affiliations:** ^1^Centre for the Research and Technology of Agro-Environmental and Biological Sciences, University of Trás-os-Montes e Alto Douro, Vila Real, Portugal; ^2^Horticulture and Product Physiology, Wageningen University, Wageningen, Netherlands

**Keywords:** growth conditions, quality indicators, phenolic compounds, new preservation technologies, breeding for quality

There is an error in the Funding statement. The correct Name for the Funder is INTERACT project—Integrative Research in Environment, Agro-Chains and Technology, no. NORTE-01-0145-FEDER-000017, in its line of research entitled ISAC-P2, co-financed by the European Regional Development Fund (ERDF) through NORTE 2020 (North Regional Operational Program 2014/2020). The authors apologize for this error and state that this does not change the scientific conclusions of the article in any way.

## Conflict of interest statement

The authors declare that the research was conducted in the absence of any commercial or financial relationships that could be construed as a potential conflict of interest.

